# Emollient prescribing formularies and guidelines in England, 2021: a cross‐sectional study

**DOI:** 10.1111/ced.15197

**Published:** 2022-05-26

**Authors:** Nana Yaa T. Amakye, Jonathan Chan, Matthew J. Ridd

**Affiliations:** ^1^ Centre of Academic Primary Care, Medical School University of Bristol Bristol UK

## Abstract

**Background:**

Emollients are a mainstay of treatment for dry skin conditions. In the UK, prescribers are usually expected to follow local National Health Service (NHS) formularies. A previous study in 2018 showed that the recommended emollients across England and Wales varied widely. Evidence has since emerged that bath additives provide no additional clinical benefit in eczema.

**Aim:**

To compare emollient formularies and guidelines in England.

**Methods:**

Clinical Commissioning Group (CCG) formularies and guidelines were identified in April–May 2021, compiled and then analysed descriptively.

**Results:**

In total, 105 CCGs, 72 emollient formularies and 47 emollient prescribing guidelines were identified. There were internal inconsistencies between formularies and their accompanying guidelines in 19% of cases. The majority (68%) of formularies/guidelines were organized using a ranking system. In total, 126 different leave‐on emollients were named. Creams and ointments were universally available and were the most recommended first‐line types. Cost was more likely than patient choice to be recommended as a criterion for selecting which emollient to prescribe. Aqueous cream was the leave‐on emollient most commonly not recommended. Nearly three‐quarters (74%) of formularies stated that bath additives should not be prescribed.

**Conclusion:**

All CCGs in England have an emollient formulary/guideline, but there is still great variability between them in their recommendations. Although the number of formularies/guidelines has reduced since 2017, there has been an increase in the total number of unique recommended leave‐on emollients. Most CCGs are no longer recommending bath emollients for eczema.

## Graphical abstract

Emollients are a mainstay of treatment for dry skin conditions. In total, 105 Clinical Commissioning Groups shared 72 emollient formularies, which recommended 126 different leave‐on emollients; 74% did not recommend bath emollients.

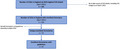

## Introduction

Emollients are universally recommended as first‐line treatment for eczema in children and adults.^1^, ^2^ They can be used as ‘leave‐on’ treatments, soap substitutes and bath additives, and are grouped according to their formulation.^3^ The majority are creams, ointments, gels or lotions, which vary in their level of greasiness, i.e. their oil/water ratios.^4^, ^5^ A minority are available as sprays (e.g. Dermamist; Alliance Pharmaceuticals, Chippenham, Wiltshire, UK) or balms (e.g. Flexitol® Heel Balm; Thornton & Ross, Linthwaite, Huddersfield, West Yorkshire, UK).^5^

In the UK, as in other countries, clinical care is supported by national (e.g. National Institute for Health and Care Excellence, Scottish Intercollegiate Guidelines Network) and local guidelines and by emollient formularies. In 2018, Chan *et al*.^6^ summarized the emollient formularies for all 216 Clinical Commissioning Groups (CCGs) and local Health Boards in England and Wales. They identified 102 formularies, which named 109 emollients and 24 bath additives, with poor consensus over which emollient should be used as first‐line treatments. Since then, the Bath Additives for the Treatment of ChildHood Eczema (BATHE) trial has been published, which demonstrated no additional benefit from bath additives for children.^7^ The Best Emollients for Eczema trial, comparing the four main types of emollients, has just reported.^4^ It is therefore an opportune time to update the 2018 review, to see what changes have been made to emollient formularies in England since 2017.

## Methods

### Data collection

A list of all the CCGs was compiled on a spreadsheet (Excel; Microsoft Corp., Redmond, WA, USA) using information from the NHS CCG directory^8^ and NHS England website^9^ in April 2021, then the emollient formularies and their associated emollient prescribing guidelines (if any) were identified.

In keeping with Chan *et al*.,^6^ we used the NICE and Oxford dictionary definitions of a formulary [‘an official list (with no explanatory information) giving details of prescribable medicines’] and a guideline (‘a general rule, principle or piece of advice’) to distinguish between the two.^10^ Each CCG website was explored first to see if a formulary could be easily identified, then keywords (‘medicine’, ‘medicines’, ‘medicine management’, ‘medicine optimisation’, ‘formulary’, ‘emollient formulary’) were entered into the website search box. If the formulary still could not be located, a search was performed on Google, using the terms ‘CCG name + emollient formulary’ and ‘CCG name + emollient guideline’.

Data were extracted by NA from the formularies/guidelines onto the Microsoft Excel template created by Chan *et al*.^6^ How the information was coded on the spreadsheet was defined in a codebook to ensure consistency. One of the authors (JC) independently checked a sample and identified discrepancies in 7% of datapoints (110 of 1500) for leave‐on emollients and 3% of datapoints (8 of 282) for bath additives. The discrepancies were discussed and most were found to be due to differing interpretations of formulary and guideline information. Most additional guidelines complemented the formulary, making data extraction straightforward. Where this was not the case, data were extracted from the most up‐to‐date document.

### Data analysis

The Excel spreadsheet was imported and analysed descriptively using STATA software (V16; StataCorp, College Station, TX, USA). All percentages were rounded to the nearest whole number unless stated otherwise.

## Results

### Number of formularies and guidelines, stratified by Clinical Commissioning Group

**Figure 1:**
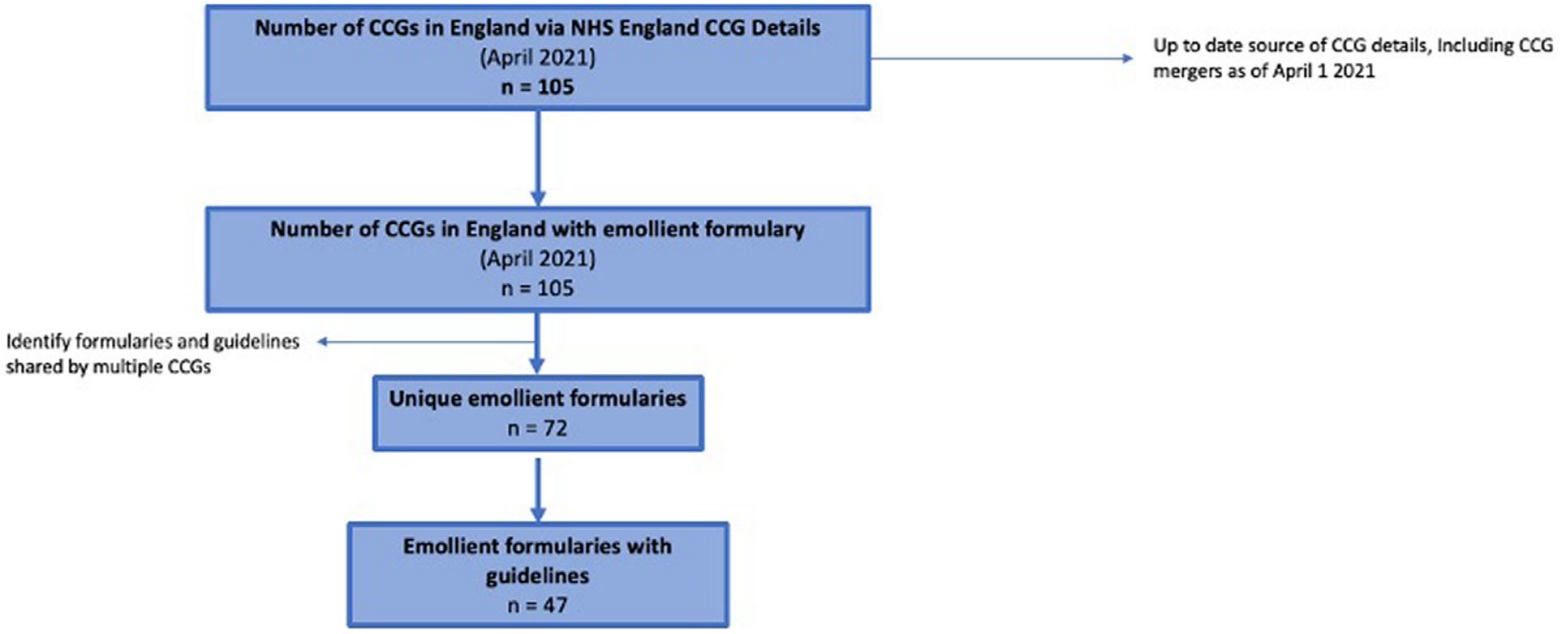
Flow chart showing the number of Clinical Commissioning Groups (CCGs), emollient formularies and emollient guidelines in England. [Colour figure can be viewed at wileyonlinelibrary.com]

**Figure 2:**
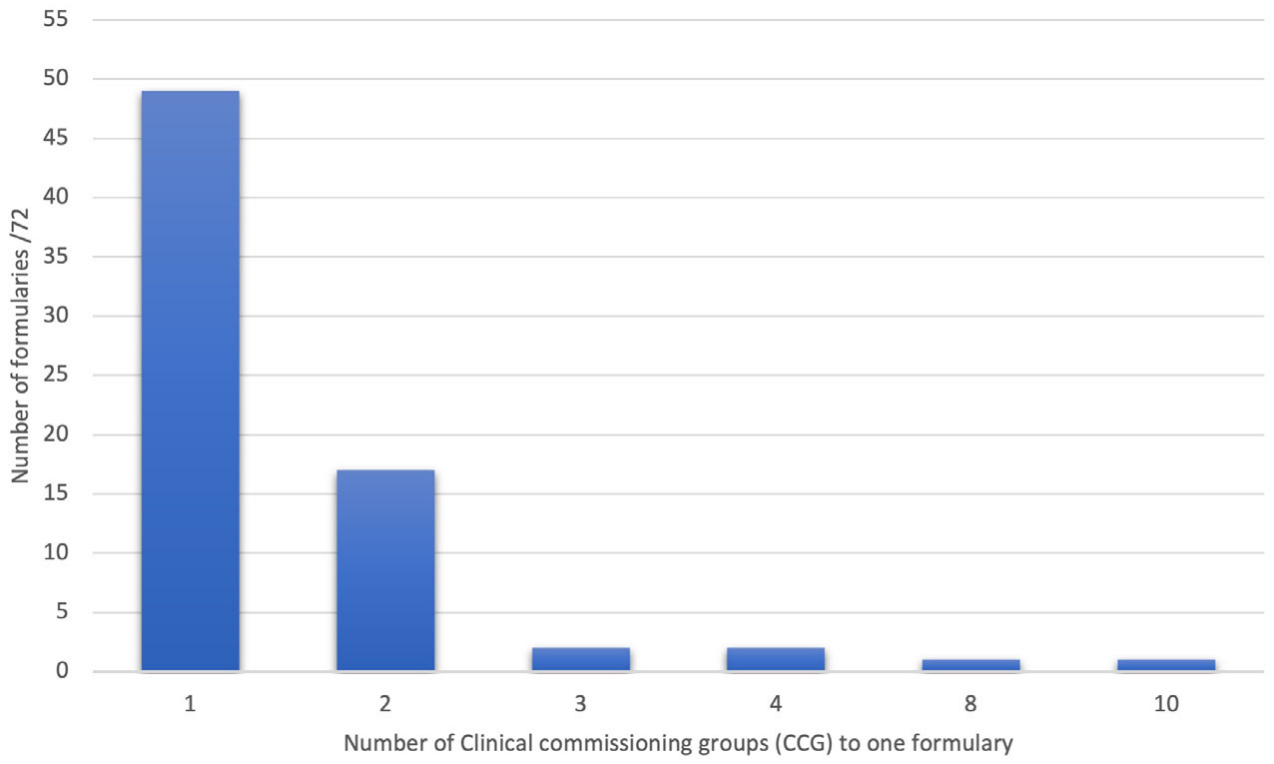
Number of Clinical Commissioning Groups (CCGs) using a single formulary. Left to right: 49 formularies 1: 1, 17 formularies 2: 1, 2 formularies 3: 1, 2 formularies 4: 1, 1 formulary 8: 1, 1 formulary 10: 1. [Colour figure can be viewed at wileyonlinelibrary.com]

In total, 105 CCGs were identified, all of which had formularies. Some formularies were shared by multiple CCGs, resulting in 72 unique emollients formularies (Fig. 1). There were 23 formularies that were shared by ≥ 2 CCGs, with the largest being the Greater Manchester joint formulary, shared by 10 CCGs (Fig. 2).

Half (36 of 72) of the CCG formularies were presented on ‘netFormulary’, a drug formulary system organized by body systems, followed by drug groups; other CCGs provided downloadable PDF or Excel documents. Most (65%; 47 of 72) of emollient formularies had additional prescribing guidelines, either as part of the formulary or as a separate document. There were inconsistencies between the formulary and the accompanying guidelines in 19% of cases (9 of 47).

Over half (57%; 41 of 72) of formularies/guidelines provided guidance on suitable quantities of emollient required for 1 week/month according to the area of the body being treated. The majority (68%; 49 of 72) of formularies/guidelines were organized using a ranking (first‐line, second‐line, etc.) system, while 32% (23 of 72) listed their emollients as a simple list. Nearly half (47%; 34 of 72) of formularies/guidelines listed emollients by formulation type. The majority (72%; 52 of 72) of formularies/guidelines mentioned one or multiple factors that prescribers should consider when selecting an emollient: 60% (43 of 72) mentioned cost, 50% (36 of 72) patient preference and 47% (34 of 72) formulation type.

### Types of emollient

#### Leave‐on emollients

In total, 126 unique types of leave‐on emollients were recommended across all the formularies and guidelines. Each formulary had an average of 23 different emollients available, with 51 being the highest number on a single formulary and 8 the lowest. All 72 formularies/guidelines recommended cream(s) or ointment(s), 94% recommended gel(s), 83% recommended lotion(s) and 79% recommended others (sprays, balms). Of the 49 formularies that used a ranking system (numbered or ‘traffic light’ systems), first‐line recommendations by type were as follows: 100% cream, 94% ointment, 69% gel, 41% lotion and 14% others.

Table 1 shows the top five most recommended creams, lotions, ointments and gels. Overall, the top five most recommended emollients were Dermol 500 lotion (59 of 72), Dermol cream (52 of 72), emulsifying ointment BP (51 of 72), Epimax cream (50 of 72) and white soft/liquid paraffin 50/50 (51 of 72). However, Dermol cream and Dermol 500 lotion both contain antimicrobials: 77% (35 of 52) and 68% (40 of 59) of formularies respectively recommended their use only in the short term in cases of eczema complicated by infection.

**Table 1:** Top 5 most recommended creams, lotions, ointments and gels.

Product	*n*/*N* (%)^a^
Cream	
Dermol	52/72 (72)
Epimax original	50/72 (69)
Zerobase	42/72 (58)
Zerocream	42/72 (58)
Balneum plus	30/72 (42)
Ointment	
Emulsifying BP	51/72 (71)
White soft/liquid paraffin 50/50	51/72 (71)
Hydromol	47/72 (65)
Epaderm	26/72 (36)
Cetraben	25/72 (35)
Gel	
Epimax isomol	36/72 (50)
Doublebase	30/72 (42)
Aproderm	11/72 (15)
Doublebase dayleve	8/71 (11)
MyriBase	7/72 (10)
Lotion	
Dermol 500	59/72 (81)
Cetraben	16/72 (22)
E45	15/72 (21)
QV	10/72 (14)
Eucerin intensive	8/72 (11)

^a^ Recommended by formularies/guidelines.

The emollient most commonly and specifically not recommended was aqueous cream (6 of 72 formularies), the most stated reason being that it contains sodium lauryl sulfate, which is known to have an adverse effect on skin. Only 16 of 72 had aqueous cream on their formularies: 2 recommended it as first line, 2 required specialist knowledge, 1 outlined its use in specific patient populations, 1 outlined a self‐care policy and 10 had no additional guidance.

#### Bath additives

The majority (74%; 53 of 72) of formularies/guidelines specifically stated that bath additives should not be prescribed, with 63% (45 of 72) of formularies justifying their recommendation: 35% referred to NHS England guidance and 29% cited research evidence (the BATHE trial). Of the 53 formularies not recommending bath additives, 7 still had one or more type of bath additive as an ‘on formulary’ item, but 5 of the 7 specified either that use should only be initiated by specialists or general practitioners (GPs) for patients with severe eczema.

In total, 29 different products were named on the 19 of 72 formularies that either recommended bath additives or listed them as ‘on formulary’ items with no added information discouraging their use. The five most recommended are shown in Table 2, and included two antimicrobial bath additives, Dermol 600 and Emulsiderm. Dermol 600 was recommended on 13 formularies; 46% (6 of 13) specified its short‐term use in those with severe/infected eczema, and 2 of these specified its use strictly in children. Emulsiderm was listed on seven formularies, two of which outlined its short‐term use in those with severe infective eczema. As an alternative to bath additives, several formularies recommended the use of soap substitutes instead. Balneum plus bath oil is a urea‐containing bath additive, which was listed on seven formularies, of which three formularies specified its use in exceptional circumstances or for severe eczema and refractory pruritis.

**Table 2:** Top 5 most recommended bath additives.

Bath additives	On formulary/recommended	
	*n*	%
Dermol 600	13	18
Hydromol	8	11
Balneum plus bath oil	7	10
Oilatum	7	10
Emulsiderm	7	10

## Discussion

This is an update of the 2018 paper by Chan *et al*.,^6^ which was the first of its kind to compare emollient formularies in England and Wales. Since 2017, there has been a reduction in the number of CCGs (from 209 to 105, as of May 2021) and formularies (from 102 to 72) in England. The benefits or consequences of shared formularies are unknown, but having fewer formularies should improve consistency in emollient prescribing and reduce variations in care. Conversely, there has been an increase in the number of accompanying guidelines, from 18 in 2018 to 47 in 2021. This could be attributable to recent literature alluding to the lack of evidence‐based emollient prescribing, encouraging CCGs to add guidance. Guidelines varied greatly, and we found that the most mentioned factor for prescribers to consider when prescribing emollients was cost (mentioned by 60% of guidelines), implying that all emollients are therapeutically equivalent, for which there is no evidence.^3^ Patient concordance with any treatment is fundamental to its effectiveness, yet the number of formularies mentioning this consideration had reduced (from 60% in 2018 to 50% in 2021). The reason for ‘internal inconsistency’ between some formularies and guidelines is unclear, but may be a product of different CCGs merging.

There has been an increase in uniquely named emollients between 2017 and 2021, from 109 to 126 different products. Creams and ointments remain the most recommended formulation types. Interestingly, the top two most recommended leave‐on emollients (Dermol 500 lotion and Dermol cream) possess antimicrobial properties. However, most formularies (81% for Dermol 500 lotion, 72% for Dermol cream) listing these qualify their use by indication. In 1998, Whitefield wrote about a new antimicrobial emollient on the market, which was liked by most patients and consequently clinicians,^11^ but two decades later, a paper published in 2019 counselled prescribers to think carefully about the use of topical antimicrobials, in order to reduce antimicrobial resistance.^12^

A notable change since 2017 is the increase in formularies/guidelines not recommending the use of bath additives. Nearly three‐quarters (74%) of the formularies/guidelines did not recommend the use of bath additives, compared with in 2017, when 82% of formularies in England and Wales recommended them. In 2018, 75% of the formularies recommending bath additives did not provide any rationale, contrasting with 63%, who now give a reason for their recommendation against them, referencing specific research evidence and/or NHS England guidance. While acknowledging that the BATHE study population were children, the NHS guidance felt able to extrapolate the findings to adolescents and adults with eczema as well.^13^

Aqueous cream BP has been used as a moisturizer since 1958,^14^ but it is now the least recommended leave‐on emollient, with 6 formularies explicitly stating that they do not recommend its use and 50 formularies not mentioning it. Most of the formularies/guidelines specifically excluding aqueous cream from their formulary referenced the MHRA UK Public Assessment Report; this report outlined findings from an audit involving 71 children (1–16 years old) with atopic eczema, which showed frequent adverse skin reactions (e.g. itchiness, redness, stinging, etc.) with aqueous cream use.^15^, ^16^

Regarding the study limitations, the scope of our review was limited to emollient formularies in England only. As the CCGs and their respective formularies are constantly changing, it is impossible to provide a completely up‐to‐date national picture of emollient formularies and guidelines. The majority of data extraction was performed by one researcher and some formularies/guidelines are open to interpretation, so imperfections in the data presented are possible.

## Conclusion

Emollient formularies and guidelines should be easily accessible and clear to follow, in order to suit time‐pressured GPs, who manage most people with dry skin conditions. There have been some notable changes since 2017, both in respect to the number of formularies and their recommendations. Although CCG mergers have led to greater sharing of fewer formularies, there was limited improvement in emollient formulary/guideline consistency across the country. The formularies and guidelines still differ greatly in their formats and level of detail, and some even contradict themselves. The bewildering number of emollients is still poorly substantiated. There has been a dramatic shift in the recommendation of bath additives in England following the BATHE trial results, and now the majority of formularies do not recommend their use. There is a need for better evidence to guide emollient prescribing and improve resource use.

**What's already known about this topic?**

- Emollients are an important treatment for dry skin conditions, but little is known about their relative effectiveness or acceptability in everyday use.
- Previous work published in 2018 identified a large number of emollients and formularies, with poor consensus over which emollients were recommended.

**What does this study add?**

- There are now fewer formularies and guidelines, which variously rank recommendations and/or list emollients by type.
- The overall number of named emollients has increased and agreement over which to try first has not improved.
- Bath additives are less likely to be recommended.

## Conflict of interest

The authors declare that they have no conflicts of interest.

## Funding

Not applicable.

## Ethics statement

Ethics approval and informed consent were not applicable.

## Data availability

The data that support the findings of this study are available from the corresponding author on reasonable request.
